# A Replicating Viral Vector Greatly Enhances Accumulation of Helical Virus-Like Particles in Plants

**DOI:** 10.3390/v13050885

**Published:** 2021-05-11

**Authors:** Eva C. Thuenemann, Matthew J. Byrne, Hadrien Peyret, Keith Saunders, Roger Castells-Graells, Inmaculada Ferriol, Mattia Santoni, John F. C. Steele, Neil A. Ranson, Linda Avesani, Juan Jose Lopez-Moya, George P. Lomonossoff

**Affiliations:** 1 John Innes Centre, Department of Biochemistry and Metabolism, Norwich Research Park, Norwich NR4 7UH, UK; hadrien.peyret@jic.ac.uk (H.P.); keith.saunders@jic.ac.uk (K.S.); roger.castells-graells@jic.ac.uk (R.C.-G.); jfcsteele@gmail.com (J.F.C.S.); 2 Astbury Centre for Structural Molecular Biology, School of Molecular and Cellular Biology, Faculty of Biological Sciences, University of Leeds, Leeds LS2 9JT, UK; M.J.Byrne@Leeds.ac.uk (M.J.B.); N.A.Ranson@leeds.ac.uk (N.A.R.); 3 Department of Chemistry and Biochemistry, University of California, Los Angeles, CA 90095, USA; 4 Centre for Research in Agricultural Genomics (CRAG, CSIC-IRTA-UAB-UB), 08193 Cerdanyola del Vallès, Spain; inmaculada.ferriol@cragenomica.es (I.F.); juanjose.lopez@cragenomica.es (J.J.L.-M.); 5 Consejo Superior de Investigaciones Científicas (CSIC), 08003 Barcelona, Spain; 6 Diamante srl. Strada Le Grazie, 15, 37134 Verona, Italy; mattia.santoni@univr.it; 7 Piramal Healthcare UK Ltd., Piramal Pharma Solutions, Earls Road, Grangemouth, Stirlingshire FK3 8XG, UK; 8 Department of Biotechnology, University of Verona, Strada Le Grazie, 15, 37134 Verona, Italy; linda.avesani@univr.it

**Keywords:** virus-like particle, overexpression, filamentous, rod-shaped, pEAQ-*HT*, pEff, *Potexvirus*, *Potyviridae*, high-resolution cryo-EM

## Abstract

The production of plant helical virus-like particles (VLPs) via plant-based expression has been problematic with previous studies suggesting that an RNA scaffold may be necessary for their efficient production. To examine this, we compared the accumulation of VLPs from two potexviruses, papaya mosaic virus and alternanthera mosaic virus (AltMV), when the coat proteins were expressed from a replicating potato virus X- based vector (pEff) and a non-replicating vector (pEAQ-*HT*). Significantly greater quantities of VLPs could be purified when pEff was used. The pEff system was also very efficient at producing VLPs of helical viruses from different virus families. Examination of the RNA content of AltMV and tobacco mosaic virus VLPs produced from pEff revealed the presence of vector-derived RNA sequences, suggesting that the replicating RNA acts as a scaffold for VLP assembly. Cryo-EM analysis of the AltMV VLPs showed they had a structure very similar to that of authentic potexvirus particles. Thus, we conclude that vectors generating replicating forms of RNA, such as pEff, are very efficient for producing helical VLPs.

## 1. Introduction

Half of known RNA plant viruses have helical structures [1]. Such viruses can be spread by vectors, mechanical damage or propagation and can cause severe symptoms in their host plant species with consequential yield losses impacting farmers and food security. Helical virus particles can be either rigid rods, for example, tobacco mosaic virus (TMV, genus *Tobamovirus*, family *Virgaviridae*), or flexuous filaments, for example, potato virus X (PVX, genus *Potexvirus*, family *Alphaflexiviridae*). In each case the coat protein subunits are arranged with helical symmetry around the genomic RNA. Since TMV and PVX infections are very high yielding, it has been possible to derive near atomic resolution structures of their particles [2,3,4]; such studies have not, to date, been possible on lower yielding viruses.

As well as being a curse from a plant pathology point of view, rod-shaped viruses are also a blessing as their particles have characteristics which lend themselves to exploitation for many bio- and nanotechnological applications, from their use as epitope carriers for the development of vaccines and diagnostics, to their use as templates for nanowire synthesis [5,6,7,8]. Most of this work has been carried out on particles produced via infection and has therefore been largely limited to those viruses, such as TMV and PVX, that are high yielding. Relying on infection to produce particles does, however, limit the scope of modifications that can be incorporated since virus functionality must be maintained. To address this, there have been attempts to make non-infectious virus-like particles (VLPs). These have involved either in vitro assembly of the viral coat on RNA or the expression and self-assembly of the viral coat protein in vivo in a variety of hosts, including plants. However, in vitro assembly is only feasible on a small scale with a virus, such as TMV, that has been extensively studied. While expression of the coat protein (CP) and self-assembly into VLPs has been very effective with isometric plant viruses [5,9,10,11,12], expression of the coat protein alone in the case of helical viruses generally results in either low yields, very heterogeneous material, or both. For example, the non-replicating pEAQ-*HT* system [13] has been used for the production of turnip mosaic virus VLPs by expression of coat protein alone [14], and tobacco mosaic virus VLPs by co-expression of coat protein with RNA bearing a TMV origin of assembly (OAS; [15]); however only relatively low yields were reported. The only exception to this was found when a replicating PVX vector was used to express the coat protein of the related potexvirus, alternanthera mosaic virus (AltMV; [16]). These results suggested that co-expression of the coat protein with an appropriate, replicating RNA might be the key to producing VLPs of helical plant viruses. The possibility that replicating RNA may be necessary for the efficient generation of helical VLPs would be consistent with our recent studies on the requirements for RNA packaging in the isometric particles of cowpea mosaic virus (CPMV; [17]) and satellite tobacco necrosis virus (STNV; [18]).

To test this hypothesis, we have compared the level of coat protein and VLP accumulation of two potexviruses, AltMV and papaya mosaic virus (PapMV) when expressed from the non-replicating pEAQ-*HT* vector and the replicating PVX-based vector pEff [19]. The results showed significantly enhanced accumulation of the coat protein and the formation of filamentous VLPs when the pEff vector was used. Furthermore, we showed that expression of the coat proteins of unrelated plant virus genera (TMV, cucumber vein yellowing virus (CVYV, genus *Ipomovirus*, family *Potyviridae*) and sweet potato feathery mottle virus (SPFMV, genus *Potyvirus*, family *Potyviridae*)) from the pEff vector leads to the efficient formation of helical VLPs. Analysis of the RNA content of the AltMV and TMV VLPs revealed that they contain vector-derived RNA sequences and Cryo-EM analysis has shown that AltMV VLPs have a structure characteristic of authentic potexviruses. Thus, we conclude that the deployment of a replicating vector, such as pEff, is an efficient approach to producing structurally relevant VLPs of helical viruses.

## 2. Materials and Methods

### 2.1. Cloning of Constructs

Genes encoding the coat proteins (CP) of helical viruses were obtained from various sources: AltMV-CP (GenBank: FJ822136), SPFMV-CP (GenBank: NC001841) and PapMV-CP (GeneBank: D13957.1) were ordered from GeneArt (Thermo Fisher Scientific GENEART GmBH, Regensburg, Germany). CVYV-CP was amplified from cloned materials corresponding to the isolate CVYV-Esp (GeneBank: MK777994) [20] kindly provided by Cecile Desbiez (INRA, Avignon, France). TMV-CP/OAS was amplified from pEAQ-*HT*-TMV-CP/OAS [15].

A Gateway cloning LR reaction with LR clonase II (Thermo Fisher Scientific, Loughborough, UK) was used according to the manufacturer’s instructions to transfer the PapMV-CP gene into pEAQ-*HT*-DEST1 [13] to yield pEAQ-*HT*-GW-PapMV-CP. The gene for AltMV-CP was amplified from a GeneArt construct with end-tailoring primers to introduce a downstream stop codon and XhoI site (Table S1), and was subsequently cloned using an existing upstream AgeI and new downstream XhoI site into pEAQ-*HT* to give pEAQ-*HT*-AltMV-CP. All aforementioned genes were amplified with end-tailoring primers to introduce an upstream AscI site and downstream XmaI site for cloning into pEff-GFP [19], to make pEff-AltMV-CP, pEff-PapMV-CP, pEff-SPFMV-CP, pEff-CVYV-CP and pEff-TMV-CP/OAS.

Plasmids were selected and propagated by transforming into *Escherichia coli* Top10 cells (Thermo Fisher Scientific). Plasmids were isolated and sequenced for confirmation. All plasmids were then transformed into *Agrobacterium tumefaciens* LBA4404 for transient expression.

### 2.2. Transient Expression in Nicotiana benthamiana

Transient expression was carried out in 3–4 week old *Nicotiana benthamiana* plants by syringe infiltration as described previously [21]. Leaves were harvested 5–7 days post infiltration (dpi) and processed fresh or frozen at −80 °C.

### 2.3. Protein Extraction and Analysis

Harvested leaf tissue was either processed as small samples consisting of 3 leaf discs taken using a cork borer (number 6, Sigma Aldrichcatalogue number Z165220, Merck Life Science UK Ltd, Gillingham, UK), or larger samples for purification. Small samples were homogenized in 300 µL of extraction buffer using a Bead Ruptor 24 (Camlab, Cambridge, UK; speed = 4, 30 s, 5 °C). Leaves infiltrated with AltMV-CP, PapMV-CP and empty vector constructs were homogenized in 10 mM Tris-HCl, pH 8.0; SPFMV-CP was extracted in 0.1 M sodium borate (pH 7.95); CVYV-CP was extracted in 40 mM Tris-HCl (pH 8.7), 50 mM Ethylenediaminetetraacetic acid (EDTA), 0.1% (v/v) Triton X-100; TMV-CP/OAS was extracted in 100 mM sodium phosphate, pH 7.0.

For analysis of crude extract, a small sample was transferred to a fresh tube and mixed at a ratio of 2:1 with loading buffer (a mixture of 3 volumes LDS sample buffer (Novex, Thermo Fisher Scientific, Loughborough, UK) and 1 volume of 2-mercaptoethanol). The remaining crude extract was clarified by centrifugation in a microcentrifuge at 2000× *g* for 10 min at room temperature. For analysis of clarified extract, a small sample was transferred to a fresh tube and mixed at a ratio of 2:1 with loading buffer. Samples were boiled for 5 min, then analyzed by gel electrophoresis on 12% (w/v) NuPAGE Bis-Tris polyacrylamide gels run with MOPS SDS running buffer (Novex, Thermo Fisher Scientific). Gels were stained using InstantBlue (Abcam, Cambridge, UK).

For Western blot analysis, gels were transferred onto nitrocellulose membrane (Invitrogen, Thermo Fisher Scientific, Loughborough, UK) using a standard wet transfer technique. Membranes were incubated in blocking buffer (5% (w/v) skimmed milk powder in phosphate buffer saline with 0.1% (v/v) Tween 20) for 30 min, then primary anti-PapMV antibody (V245-C2, NANO Diagnostics LLC, Fayetteville, USA) was added at 1:2000 dilution and incubated on a shaker at room temperature overnight. Both AltMV and PapMV react with this anti-PapMV antibody. Secondary goat anti-rabbit horseradish peroxidase (HRP) antibody (Cat. ab6721, Abcam, Cambridge, UK) was diluted 1:10,000 in blocking buffer. Membranes were developed using chemiluminescent substrate (Immobilon, Merck KGaA, Darmstadt, Germany) and chemiluminescence was detected using an ImageQuant LAS 500 (GE Healthcare UK Ltd., Chalfont St Giles, UK).

### 2.4. Purification of AltMV VLPs

Leaf tissue was homogenized in 3 volumes of 10 mM Tris-HCl, pH 8.0 using a laboratory blender (Waring Lab, Torrington, USA) at 4 °C. Crude extract was filtered through 2 layers of Miracloth (Merck KGaA, Darmstadt, Germany), then clarified at 2000× *g*, 15 min, 4 °C. Clarified supernatant was acidified by adding 1/12th volume of 0.2 M citrate buffer, pH 4.0, mixed by inversion, then incubated for 15 min at room temperature. The acidified sample was again clarified (2000× *g*, 15 min, 4 °C), then mixed with 20% PEG 6000 (w/v), 1 M NaCl at a ratio of 3:1 (sample:PEG solution) and incubated at 4 °C for 2 h or overnight with gentle agitation. The PEG precipitant was pelleted (15,000× *g*, 15 min, 4 °C), then resuspended in a small volume of 10 mM Tris-HCl, pH 8.0, and finally clarified (4000× *g*, 15 min, 4 °C). This sample was used for Northern blot analysis of pEff-AltMV-CP.

For Northern blot analysis of pEAQ-*HT-*AltMV-CP, the sample was further concentrated and polished using a second pH 4 step: Sample volume was reduced to 1/10th by spin filtration (Amicon 100 kDa MWCO filter, Merck KGaA, Darmstadt, Germany). Then, a second pH 4 step was performed by addition of 1/12th volume of 0.2 M citrate buffer, pH 4.0, inversion, and immediate clarification (2000× *g*, 15 min, 4 °C). The supernatant was buffer exchanged on a PD-10 desalting column (GE Healthcare UK Ltd., Chalfont St Giles, UK) equilibrated with 10 mM Tris-HCl, pH 8.0. Finally, the sample was concentrated by spin filtration (Amicon 100 kDa MWCO filter, Merck KGaA, Darmstadt, Germany).

For cryo-EM analysis, the pEff-AltMV-CP sample was further concentrated and purified by centrifugation through a sucrose cushion: The sample (after PEG precipitation, resuspension and clarification as detailed above) was concentrated by spin filtration (Amicon 100 kDa MWCO filter, Merck KGaA, Darmstadt, Germany). The concentrated sample was applied to a 400 µL sucrose cushion (25% sucrose (w/v) in 10 mM Tris-HCl) and centrifuged at 36,000 rpm for 2 h at 4 °C on a TLS-55 rotor (Beckman Coulter UK Ltd, High Wycombe, UK). The sucrose step was collected, buffer exchanged on a PD-10 desalting column equilibrated with 10 mM Tris-HCl, pH 8.0, and concentrated by spin filtration as above.

### 2.5. Purification of TMV VLPs

Tobacco mosaic virus VLPs were prepared by PEG precipitation and differential centrifugation as described by Saunders and Lomonossoff [15].

### 2.6. Northern Blots

RNA was analyzed by Northern blot as described by Kruse et al. [17]. In short, RNA extracted from equal amounts of particles (as measured by protein content using a Pierce BCA protein assay according to manufacturer instructions (Thermo Fisher Scientific) by phenol:chloroform extraction and lithium chloride precipitation was analyzed by electrophoresis under denaturing conditions in formaldehyde-containing agarose gels, in the presence of ethidium bromide [22]. For Northern blotting, the RNA was transferred from the agarose gel to a positively charged nylon membrane and probed with digoxigenin (DIG)-labelled RNA probes specific for the sequence of interest. The probes were synthesized from PCR fragments using the DIG starter labelling kit (Roche, Merck KGaA, Darmstadt, Germany) and were detected on the Northern blot membrane by alkaline phosphatase-conjugated anti-DIG antibody (Roche, Merck KGaA, Darmstadt, Germany). The signal was detected colorimetrically using BCIP (5-bromo-4-chloro-3-indolylphosphate) substrate tablets (Merck KGaA, Darmstadt, Germany).

### 2.7. Transmission Electron Microscopy (TEM)

Clarified extracts and purified samples were adsorbed onto 400-mesh carbon-coated copper grids (EM Resolutions, Sheffield, UK) for 30 s, then rinsed with deionized water before staining with 2% (w/v) uranyl acetate for 15–30 s. Grids were imaged using a Talos F200C TEM fitted with a Gatan OneView camera.

### 2.8. Cryo Electron Microscopy

Cryo-EM grids were prepared by first glow discharging for 30 s (easiGlow, Ted Pella). Furthermore, 3 uL of AltMV VLP suspension was applied to 400 mesh lacey carbon grids, with an ultrathin carbon support. The sample was blotted immediately, and frozen in liquid ethane cooled by liquid nitrogen at 95% humidity and 4 °C using an FEI Vitrobot mark IV (Thermo Fisher Scientific). Data was collected on a ThermoFisher Titan Krios (Astbury Biostructure Laboratory, University of Leeds) electron microscope at 300 kV, with a total electron dose of 74.26 ^e−^/Å^2^ at 75,000× magnification. Each movie had a total exposure of 1.5 s across 59 frames. Data collection parameters are shown in Table S1. Data collection was carried out as described in [23].

#### 2.8.1. Image Processing

Image processing was carried out using RELION 3.1. Motion correction of movies was carried out with MOTIONCOR2 and the contrast transfer function of each determined with gCTF. Particles were picked manually as ‘start and end’ coordinates in RELION 3.1 and extracted as helical segments. In total 388,226 segments were extracted. Two-dimensional classification with helical parameters was used to obtain the straightest of the extracted helical segments. A featureless cylinder was used to generate an initial model in 3D classification. The output model allowed for rough estimation of the helical parameters of rise and twist. Further 3D classification with local searches allowed for more accurate estimation of helical parameters, and subset selection of the best particle segments. An initial round of refinement gave a structure at 3.63 Å resolution. Per particle contrast transfer function (CTF) refinement and Bayesian particle polishing were carried out on the resulting particle stack comprising 32,108. Subsequent 3D refinement yielded a structure with an axial rise of 3.96 Å and a twist of 41.11° to a nominal resolution of 3.46 Å prior to sharpening. Post processing in RELION 3.1 yielded a map with a final resolution of 3.3 Å. The final resolution was determined using the ‘gold standard’ Fourier shell correlation (FSC = 0.143) criterion.

#### 2.8.2. Model Building

The initial molecular model for AltMV was built using the SWISS model homology modelling server, with the potato virus X capsid protein serving as a template (PDB 6R7G). This model was then fitted into the final post processed map using UCSF chimera. The model was then accurately fitted to the density map and modified using COOT. Iterative rounds of model building were carried out using COOT and Phenix real space refine, respectively. MolProbity was used for model validation.

## 3. Results

### 3.1. Potexvirus VLPs Are Efficiently Expressed by the Potexvirus-Based pEff Vector

**Figure 1:**
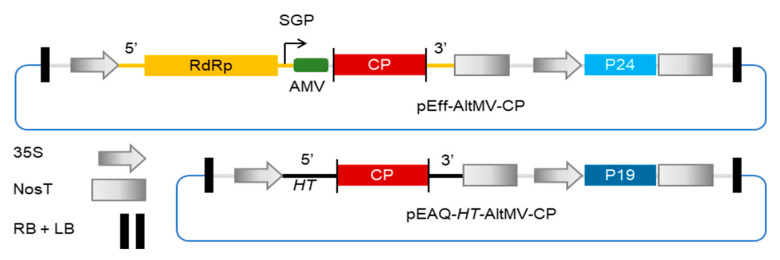
Schematic diagram of constructs used to express the AltMV CP. Both constructs have the same binary vector backbone (pEAQ, blue line) but differ in the way in which inserted coat protein (CP) sequence is expressed. Transcription from the 35S promoter of pEff results in the production of potato virus X (PVX) replicons, with expression of CP occurring via a subgenomic mRNA from the subgenomic promoter (SGP) on the PVX replicon. Translation is enhanced by the presence of the 5′ UTR from alfalfa mosaic virus (AMV) upstream of the CP and the P24 suppressor of gene silencing on the T-DNA. For pEAQ, transcription results in hyper-translatable (*HT*) mRNA with 5′ and 3′ UTRs from cowpea mosaic virus (CPMV) and expression levels are enhanced by the presence of the P19 silencing suppressor on the T-DNA.

**Figure 2:**
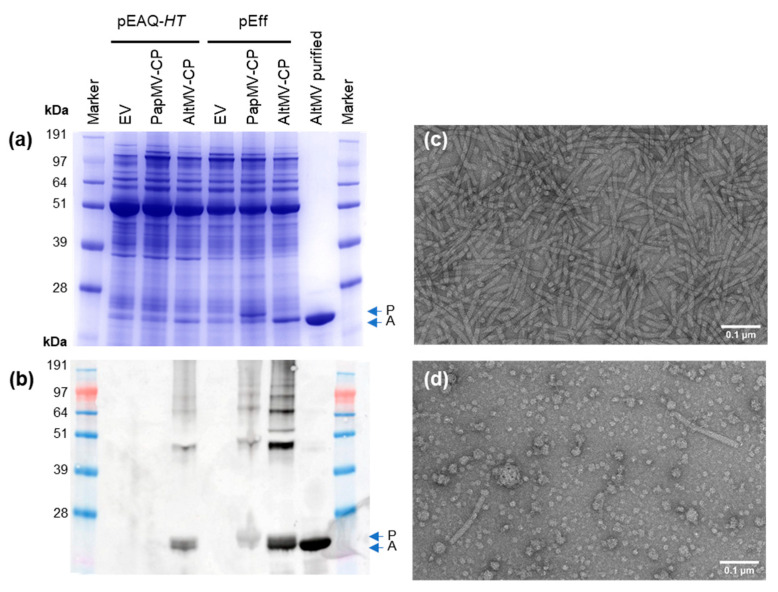
Comparison of expression of potexvirus coat proteins from pEAQ-*HT* and pEff. Analysis of clarified protein extract by InstantBlue-stained SDS-PAGE (**a**) and whole protein extract by anti-PapMV Western blot (note that anti-PapMV antibody has high affinity for both PapMV-CP and AltMV-CP) (**b**). Samples were from the same infiltration experiment. Transmission electron microscopy (TEM) images of pEff-AltMV-CP after purification (**c**), and pEff-PapMV-CP particles in clarified extract (**d**). EV: empty vector control; AltMV purified: virus-like particles (VLPs) of pEff-AltMV-CP purified using acid treatment and PEG precipitation. Blue arrows show location of bands for PapMV-CP (P) and AltMV-CP (A).

To investigate whether the use of a replicating vector could enhance the production of filamentous VLPs, we compared expression of two potexvirus coat proteins from AltMV (AltMV-CP) and PapMV (PapMV-CP), using pEAQ-*HT* or pEff. (Figure 1). Both pEAQ-*HT* and pEff constructs were infiltrated into *N. benthamiana* leaves and coat protein accumulation was assessed by SDS-PAGE analysis of protein extracts followed by either InstantBlue staining or Western blot analysis (Figure 2). Expression of pEff-PapMV-CP caused severe necrosis to develop in leaf tissue at 7 dpi though this was not the case with either the empty pEff vector or pEff-AltMV-CP (Figure S1). Expression of pEAQ-*HT*-PapMV did not cause necrosis.

SDS-PAGE of clarified protein samples followed by staining with InstantBlue revealed the presence of bands corresponding to the sizes expected for PapMV and AltMV CPs when pEff was used for expression; no such bands were evident with pEAQ-*HT* (Figure 2a). To examine whether any CP was made when pEAQ-*HT* was used and to confirm the identity of the bands in Figure 2a, Western blot analysis was carried out on total protein extracts from infiltrated leaves using an anti-PapMV polyclonal antibody which detects both PapMV-CP and AltMV-CP. Accumulation of PapMV-CP was not detectable when expressed from pEAQ-*HT*, but was evident when expressed using pEff (Figure 2b). By contrast, AltMV-CP was detected from both pEAQ-*HT* and pEff constructs (Figure 2b), albeit at a higher level with pEff. To assess the ability of AltMV-CP to form VLPs, particles present in the extracts were partially purified by reducing the pH followed by PEG precipitation. The yield of assembled, purified AltMV-CP was far higher for pEff (0.85 mg/g fresh weight tissue (FWT)) than pEAQ-*HT* (less than 1 µg/g FWT), indicating that most of the AltMV-CP produced using pEAQ-*HT* cannot be purified by the standard methods used for potexviruses. This could result from a failure of the pEAQ-expressed material to form stable VLPs, or from any particulate material being insoluble and therefore lost during the initial stages of purification. Transmission electron microscopy of purified pEff-AltMV-CP revealed numerous assembled particles (Figure 2c). Particles were also found in clarified extracts of pEff-PapMV-CP (Figure 2d). Our findings show that the PVX-based vector pEff is more efficient than pEAQ-*HT* at producing potexvirus VLPs.

### 3.2. VLPs from Other Families of Helical Virus Can Be Produced to High Level Using the pEff Vector

**Figure 3:**
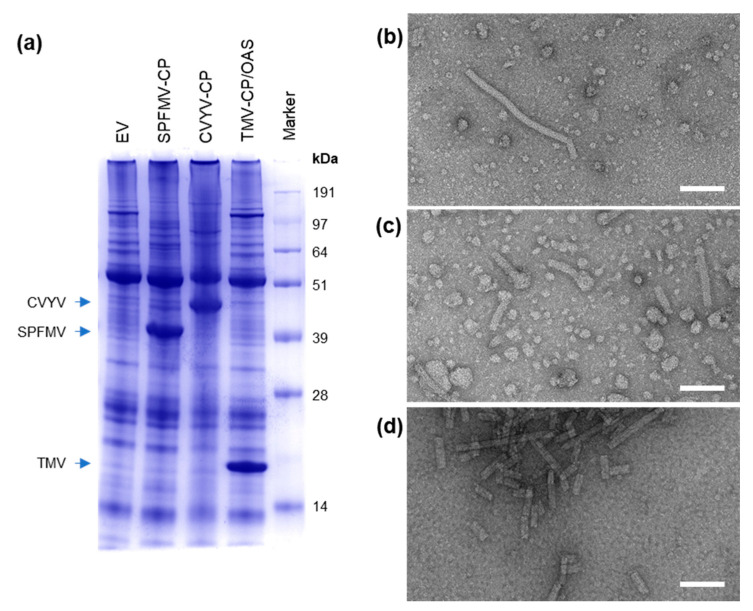
pEff allows for high-level production of filamentous VLPs from different plant virus families. Analysis of crude protein extract by Coomassie-stained SDS-PAGE (**a**). TEM images of particles from clarified extracts of pEff-SPFMV-CP (**b**), pEff-CVYV-CP (**c**) and partially purified pEff-TMV-CP/OAS (**d**). Scale bars 100 nm.

To investigate whether pEff could be a useful tool for the production of VLPs from other filamentous virus families, we inserted the coat proteins of sweet potato feathery mottle virus (SPFMV, family *Potyviridae,* genus *Potyvirus*), cucumber vein yellowing virus (CVYV; family *Potyviridae*, genus *Ipomovirus*) and tobacco mosaic virus (TMV; family *Virgaviridae*, genus *Tobamovirus*) into the pEff vector. The expression of these three viral coat proteins had previously been attempted using pEAQ-*HT* constructs and yielded no or low levels of detectable CPs for SPFMV and CVYV, respectively, and undetectable levels of particle accumulation in the case of TMV unless RNA containing the TMV OAS was co-expressed [15]. Expression of pEff-CVYV-CP caused severe necrosis to develop in leaf tissue at 7 dpi (Figure S1), whereas leaf tissue expressing pEff-SPFMV-CP or pEff-TMV-CP/OAS appeared healthy. We found that in all three cases, high levels of CP were produced and detected by InstantBlue-staining of crude extracts analyzed by SDS-PAGE (Figure 3a). The identities of the proteins were confirmed by mass spectrometry of a tryptic digest (SPFMV), Western blot (CVYV; Figure S2) or comparison with known samples in the case of TMV [15]. Filamentous particles were observed in crude extract or partially purified samples (Figure 3b–d). Purification of pEff-TMV-CP/OAS yielded 2 mg purified TMV-CP per gram FWT, an approximately 2000-fold increase in the yield obtained with pEAQ-*HT*. Yields of SPFMV-CP and CVYV-CP in leaf tissue are estimated to be similar to that of TMV-CP, judging by the similarity of band intensities in crude extract (Figure 3a). The findings show that pEff is an efficient vector for expression of coat proteins and assembled VLPs of a diverse range of filamentous plant viruses, not limited to the family and genus from which the PVX-based vector is derived.

### 3.3. pEff-Produced AltMV and TMV VLPs Encapsidate RNA

**Figure 4:**
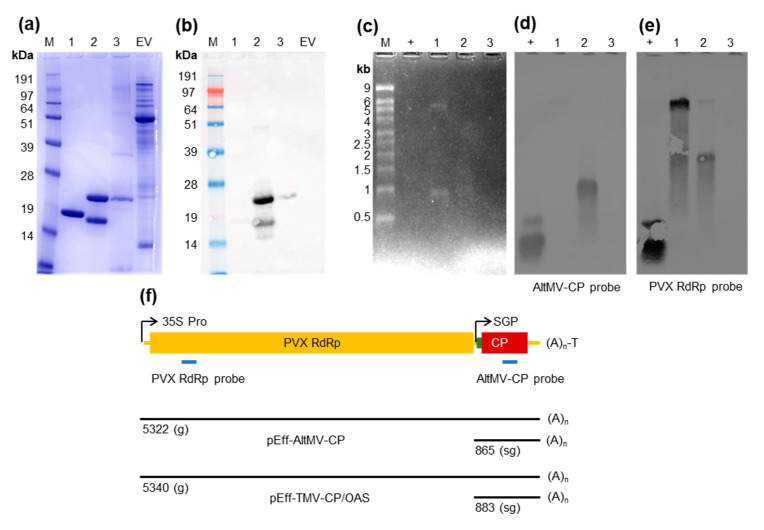
VLPs made with pEff package specific RNAs. VLPs were purified from three constructs: (1) pEff-TMV-CP/OAS; (2) pEff-AltMV-CP and (3) pEAQ-*HT*-AltMV-CP. Equal amounts of each particle preparation, as judged by protein content, were analyzed by Coomassie-stained SDS-PAGE (**a**) and anti-PapMV western blot (**b**). RNA was extracted from 50 µg of each preparation and run on duplicate agarose gels for Northern blotting. Ethidium bromide-stained RNA gel (**c**). Northern blots were probed with AltMV-CP probe (**d**) and PVX RdRp probe (**e**). Schematic diagram (**f**) showing genetic elements (relative lengths to scale), probe binding sites (blue) of pEff-AltMV-CP and PVX RdRp probes, and expected lengths of full-length ‘genomic’ RNA (g) and subgenomic RNA (sg). M: marker; EV: empty vector control clarified extract; +: positive control PCR product (DNA) of AltMV-CP or PVX Rep containing probe binding site.

In order to investigate whether particles produced using pEff and pEAQ-*HT* contain RNA, we expressed and purified AltMV VLPs using pEAQ-*HT*-AltMV-CP and pEff-AltMV-CP, and TMV-CP/OAS VLPs using pEff-TMV-CP/OAS (Figure 4a). Despite processing over 10-fold more leaf tissue for pEAQ-*HT*-AltMV-CP (150 g) than for pEff-AltMV-CP (13 g), the yield of purified AltMV-CP was lower from pEAQ-*HT*. Furthermore, the material contained additional proteins, presumably of host origin, making accurate quantitation difficult. The AltMV-CP in pEff-produced VLPs was present in two forms, full-length (22 kDa) and truncated (18 kDa), whilst pEAQ-produced VLPs contained only the full-length form (Figure 4a,b). Both AltMV and TMV samples derived from pEff have an absorbance peak at 260 nm, indicating presence of nucleic acid (Figure S3). This peak is not observed in the pEAQ-*HT*-AltMV-CP sample. Protein concentration was quantified using a BCA assay and RNA was extracted from 50 µg of each preparation. More RNA was extracted from pEff-AltMV-CP (426 ng) and pEff-TMV-CP/OAS (527 ng) than pEAQ-*HT*-AltMV-CP (118 ng). RNA samples were split in two and loaded in duplicate sets on denaturing agarose gels for electrophoresis. Ethidium bromide-stained gels were imaged (Figure 4c) and revealed prominent bands of approx. 1 kb in both pEff-AltMV-CP and pEff-TMV-CP/OAS lanes, and a band of approx. 5–6 kb for pEff-TMV-CP/OAS. No bands were observed in the sample derived from pEAQ-*HT*-AltMV-CP. The presence of distinct bands from the preparation of total RNA of purified particles suggests that the pEff-produced particles encapsidate specific RNA molecules.

To confirm that the bands observed in the stained gel were derived from the replicating pEff constructs, Northern blots were performed using two different RNA probes: one against the AltMV-CP gene (AltMV-CP probe), and one against the PVX replicase gene (PVX RdRp probe). When the AltMV-CP probe was used, a strong band at approx. 1 kb was observed in the RNA extracted from the pEff-AltMV-CP VLPs (Figure 4d). This is consistent with the expected length of pEff-AltMV-CP subgenomic RNA (>816 nt, measured to the start of the poly(A) tail, (Figure 4f). No equivalent signal was observed with RNA extracted from the pEAQ-*HT*-AltMV-CP VLPs suggesting that the mRNA is not encapsidated. Unsurprisingly, no signal was detected in RNA extracted from the pEff-TMV-CP/OAS VLPs since the sequence encoding the AltMV CP is not present.

When the PVX RdRp probe was used, a strong band of approx. 5–6 kb was observed for pEff-TMV-CP/OAS RNA, and a weaker band of similar size was observed for pEff-AltMV-CP RNA (Figure 4e). These bands are consistent with the expected length of ‘genomic’ PVX RNA from these constructs (>5340 nt, >5318 nt, respectively, measured from the start of PVX 5′ UTR to the start of the poly(A) tail, Figure 4f). Both pEff constructs also produce a band at approx. 2 kb, the origin of which is unclear. As it is detected by the RdRp probe, but not the AltMV CP probe, it must be derived from the 5′ portion of the vector-derived RNA. No signal is observed in the RNA extracted from the pEAQ-*HT*-AltMV-CP material. These results show that pEff-produced AltMV and TMV contain RNA and that most of the encapsidated RNA is specifically derived from the replicating pEff constructs.

### 3.4. The Structure of pEff-Produced AltMV VLPs Resembles Authentic Potexvirus Virions

**Figure 5:**
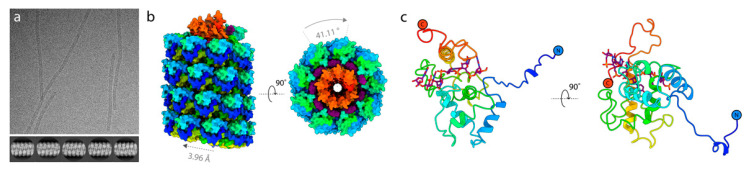
The cryo-EM structure of AltMV VLP. (**a**) Top: A representative micrograph of AltMV filaments in vitreous ice (magnification 75,000×) and bottom: 2d class averages of AltMV particle segments. (**b**) The structure of AltMV shown in surface representation with the coat protein colored from N-terminus (blue) to C-terminus (red). RNA is colored purple. Labels denote the determined helical parameters used in 3D refinement. (**c**) The AltMV coat protein atomic model shown in cartoon representation.

To determine whether the VLPs expressed from pEff are accurate mimics of the original virus structure, the structure of AltMV VLP was determined to 3.3 Å resolution (Figure 5, Figure S4). This revealed a left-handed helix, ~120 Å in diameter, comprising 8.8 coat proteins per helical turn with a helical twist of 41.1 degrees and a rise of 3.96 degrees. The coat protein of AltMV resembles those of other flexuous filamentous viruses for which structures have already been elucidated, including PVX [3], PepMV [24], BaMV [25], WMV [26], TuMV [27] and PapMV [28]. The density map was of sufficient quality to allow for unambiguous modelling of residues Phe5 to Leu203, and for the placement of five RNA residues per individual coat protein. Like PVX, the AltMV coat protein comprises three domains, domain I (residues 5–26), domain II (residues 27–155) and domain III (residues 156–203). Domain I takes the form of an extended arm-like structure, embracing the neighboring coat protein on the outer surface of the virion. Domain II makes up the core of the coat protein fold and comprises four alpha helices. Domain II is connected at its C-terminus via a long loop (107–155) to Domain III which comprises a single alpha helix (residues 156–177), and a C-terminal extension (residues 178–203). The interface between domains II and III forms a crevice within which the RNA binds, whilst the C-terminal extension of domain III extends into the filament lumen and interacts with the coat proteins eight and nine places ahead in the helical array.

## 4. Discussion

In this study, we have shown that viral coat protein expression using the replicating PVX-based vector, pEff, results in exceptionally high yields of helical VLPs, not just of related potexviruses but of other helical virus families whose study and application will benefit from such an efficient production system. This effect is not simply due to high levels of protein expression from pEff, as previous studies comparing expression levels of other proteins using pEff and pEAQ-*HT* showed that they gave broadly similar levels [19]. By using spectrophotometry, Northern blotting and cryo-EM, we have shown clearly that pEff-produced AltMV-like particles contain RNA derived from replication of the pEff construct. This is in contrast to findings of Tyulkina et al. [16] who reported that a similar PVX-based vector produced empty virus-like particles, though the data (absorption spectrum) was not presented. The discrepancy between the two results may lie in the fact that some VLPs from AltMV, unlike PapMV, can be produced in the absence of replicating RNA and that these VLPs are RNA-free. Thus, the precise methods of particle preparation may determine the proportion of RNA-containing material. Since the RNA produced by pEff-TMV-CP/OAS contained an OAS, it is also not surprising that VLPs were formed, though the efficiency was dramatically higher than previously reported [15]. Our data suggests that the presence of an abundant, nascent RNA scaffold at the site of assembly aids either the assembly, stability, or both, of the resulting VLPs, a theory that has also been proposed for STNV [18].

Whilst it is, perhaps, not surprising that the presence of a PVX-based RNA scaffold boosts the production of VLPs of the same genus (AltMV and PapMV) or with RNA containing a known OAS, we had not expected the same effect for other virus families where no such sequences have been identified. Previous attempts to produce VLPs of SPFMV and CVYV (both members of the large *Potyviridae* family) using pEAQ-*HT* had failed. *Potyviridae* CP have been shown to assemble in vitro in the presence or absence of RNA [29] and in vivo in different heterologous expression hosts [30]. More recently, González-Gamboa et al. [14] showed for the first time the production of empty potyvirus-like particles in *N. benthamiana* by expression of turnip mosaic virus CP using pEAQ-*HT*; however, their reported yields were low (10 μg/g FWT). Using pEff to produce coat proteins of SPFMV and CVYV results in much higher yields (approx. 2 mg/g FWT) and stably assembled VLPs.

Using high-resolution cryo-EM reconstruction, we have shown as a proof-of-concept that pEff-produced AltMV VLPs assemble into authentic structures resembling the recently published structure of PVX virions [3]. The high resolution of 3.3 Å achieved for pEff-produced AltMV VLPs compares favorably to the lower resolution of 8 Å recently published for pEAQ-*HT* produced turnip mosaic virus VLPs [27]. This could be due to the stabilizing effect of the RNA inside pEff-produced particles. It is highly likely that the same is true for VLPs of other viruses thus, in future, this technology can be used to solve the structures of more elusive helical plant viruses. Furthermore, VLPs which resemble the authentic virus but are non-infectious provide a safe tool for the study of the biological properties of virus particles, such as their role in insect transmission.

The need for a nucleic acid scaffold for the efficient production of VLPs of helical viruses parallels the situation found with the ssDNA-containing geminiviruses [12,31]. As with the helical viruses reported here, expression of the coat protein alone does not result in the production of any particles and the presence of replicating ssDNA is essential. Furthermore, the precise morphology of the particles is governed by the length of the replicating DNA [12,31]. Though we have not investigated this in the current study, it is probable that the length of the helical VLPs could also be controlled by varying the length of the replicating RNA.

Since the production platform we describe here does not require the resulting VLPs to be infectious, it may be possible to modify the particles more extensively than previously possible by modifying either the coat protein subunits, incorporating designer RNAs, or both. For example, PapMV, expressed in *Escherichia coli* and assembled on ssRNA in vitro, has been studied recently for its potential use as vaccine epitope carrier and adjuvant [32,33,34]. The use of plants as a production system may have benefits over bacterial expression, especially in the case of the display of peptides which may benefit from expression in a eukaryotic host. TMV particles, in particular, have been widely developed as a tool in nanoelectronics, batteries and biosensors [35]. Such applications require large-scale production, which we now show is possible using pEff. Furthermore, the ability to encapsidate designer RNA, as shown in principle with TMV [15], opens up possibilities for the development of robust delivery vehicles for RNAs, such as RNA vaccines, which have been shown to benefit from protection within a VLP [36] and may further benefit from the well-known adjuvant effect of the viral shell [37,38].

## Data Availability

Coordinates are deposited in the Protein Data Bank under accession code 7OG6. Cryo-EM reconstructions are deposited in the EM Data Bank under accession code EMD-12879. All reagents and relevant data are available from the authors upon request.

## Supplementary Materials

The following are available online at https://www.mdpi.com/article/10.3390/v13050885/s1, Figure S1: Some pEff constructs cause necrotic symptoms in leaf tissue; Figure S2: Detection of CVYV CP by Western blot; Figure S3: Absorbance spectra of purified particles; Figure S4: Fourier shell correlation plot for the cryo-EM structure of AltMV. Table S1: Primers used in cloning and RNA probe synthesis; Table S2: Cryo-EM data collection, refinement and validation statistics.
