# Supplementary material for: A Replicating Viral Vector Greatly Enhances Accumulation of Helical Virus-Like Particles in Plants
Source: Viruses. 2021 May 11;13(5):885. doi: 10.3390/v13050885 (PMC8150850; doi:10.3390/v13050885)
Supplement: Supplementary file 1 [file viruses-13-00885-s001.zip › viruses-1193296-supplementary.pdf]

Supplementary Material

A Replicating Viral Vector Greatly Enhances Accumulation of Helical Virus-Like Particles in Plants

Eva C. Thuenemann[1,*], Matthew J. Byrne[2], Hadrien Peyret[1], Keith Saunders[1], Roger Castells-Graells[1,3], Inmaculada Ferriol[4], Mattia Santoni[5], John F. C. Steele[1,6], Neil A. Ranson[2], Linda Avesani[7], Juan José López-Moya[4] and George P. Lomonossoff[1,*]

[1] Department of Biochemistry and Metabolism, John Innes Centre, Norwich Research Park, Norwich NR4 7UH, UK; eva.thuenemann@jic.ac.uk (E.C.T.); hadrien.peyret@jic.ac.uk (H.P.); keith.saunders@jic.ac.uk (K.S.); george.lomonossoff@jic.ac.uk (G.P.L.)

[2] Astbury Centre for Structural Molecular Biology, School of Molecular and Cellular Biology, Faculty of Biological Sciences, University of Leeds, Leeds, UK

[3] Present address: Department of Chemistry and Biochemistry, University of California, Los Angeles, CA 90095, USA

[4] Centre for Research in Agricultural Genomics (CRAG, CSIC-IRTA-UAB-UB), 08193 Cerdanyola del Vallès, Barcelona, Spain

[5] Diamante srl. Strada Le Grazie, 15, 37134 Verona, Italy

[6] Present address: Piramal Healthcare UK Ltd., Piramal Pharma Solutions, Earls Road, Grangemouth, Stirlingshire, Scotland, UK, FK3 8XG

[7] Department of Biotechnology. University of Verona. Strada Le Grazie, 15, 37134 Verona, Italy.

* Correspondence: eva.thuenemann@jic.ac.uk (E.C.T.); george.lomonossoff@jic.ac.uk (G.P.L.)

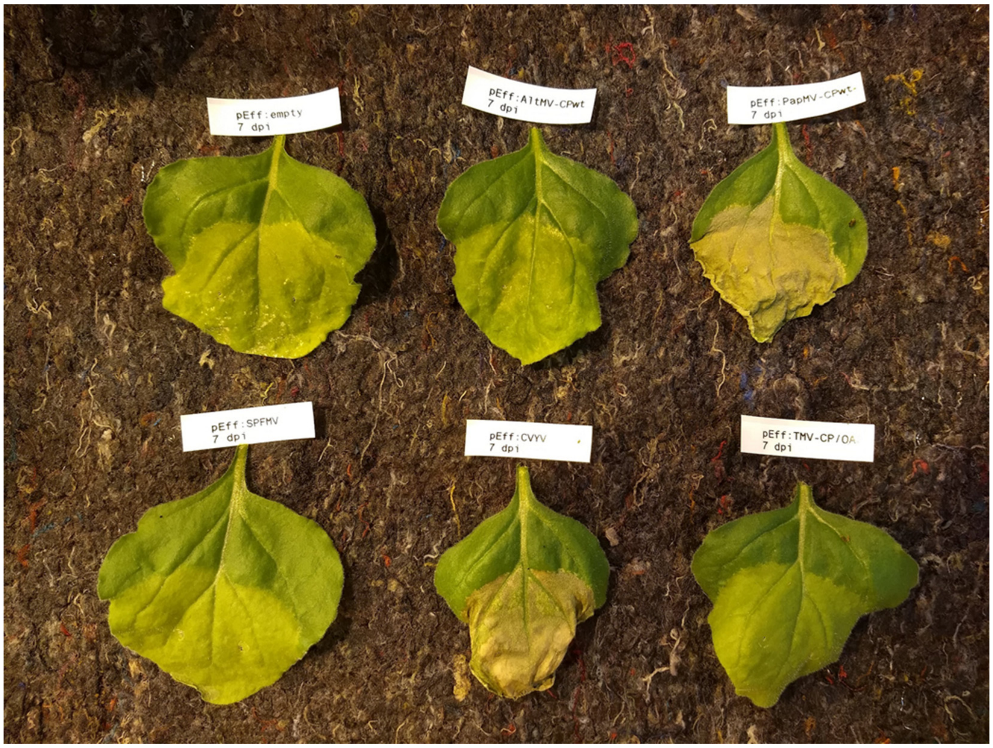

Figure S1: Some pEff constructs cause necrotic symptoms in leaf tissue. Leaves were harvested and photographed at 7 dpi. Top row left to right: pEff-empty, pEff-AltMV-CP, pEff-PapMV-CP. Bottom row left to right: pEff-SPFMV-CP, pEff-CVYV-CP, pEff-TMV-CP/OAS.

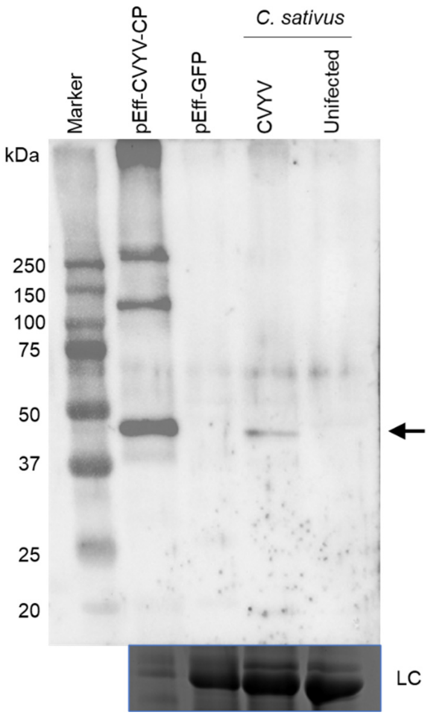

Figure S2: Detection of CVYV CP by western blot. Analysis of whole protein extract by SDS-PAGE and western blot with specific anti-CVYV antibodies [38]. Primary antibody was used at a 1:2,000 dilution and incubated at room temperature for 1h, followed by 30 min incubation with a secondary donkey anti-rabbit HRP antibody (Cytiva, GE Healthcare Life Sciences) diluted 1:10,000 in blocking buffer. Protein extracts after the Marker (first lane, kDa indicated on the left side) corresponded to samples at six-dpi of *N. benthamiana* plants agroinfiltrated with pEff-CVYV-CP (second lane) and pEff-GFP (third lane), side by side with samples of cucumber (*Cucumis sativus*) plants, infected with CVYV-Esp after sixteen days post-inoculation and an uninfected control (labelled with CVYV and Uninfected, respectively). Arrow shows the expected mobility for the monomeric CVYV-CP. The loading control below (indicated as LC) corresponds to a Coomassie-stained SDS-PAGE showing the large subunit of Rubisco protein.

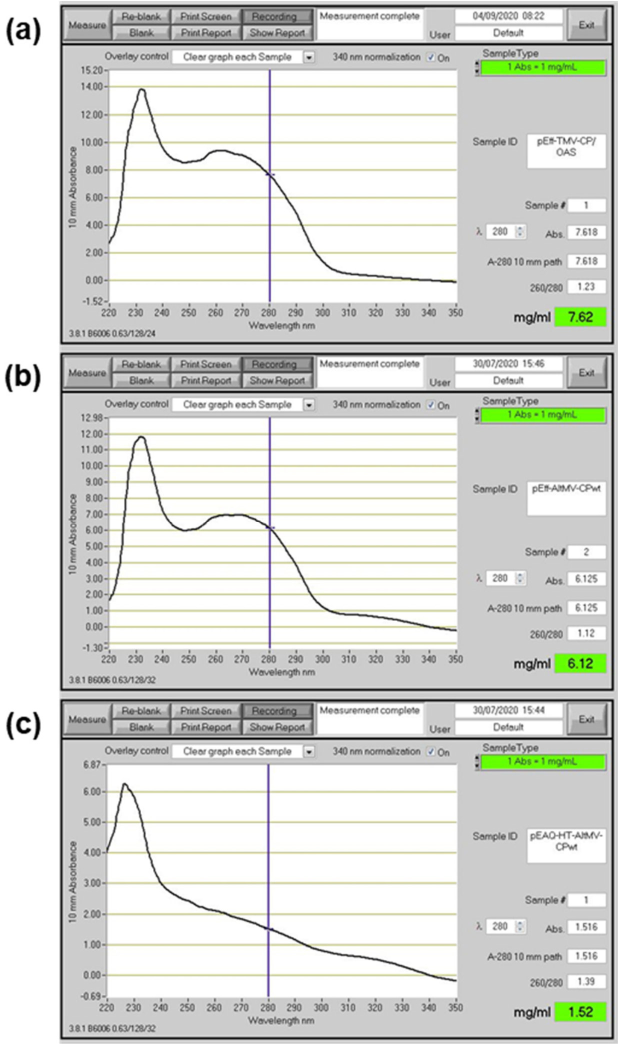

Figure S3: Absorbance spectra of purified particles from pEff-TMV-CP/OAS (**a**), pEff-AltMV-CP (**b**) and pEAQ-*HT*-AltMV-CP (**c**).

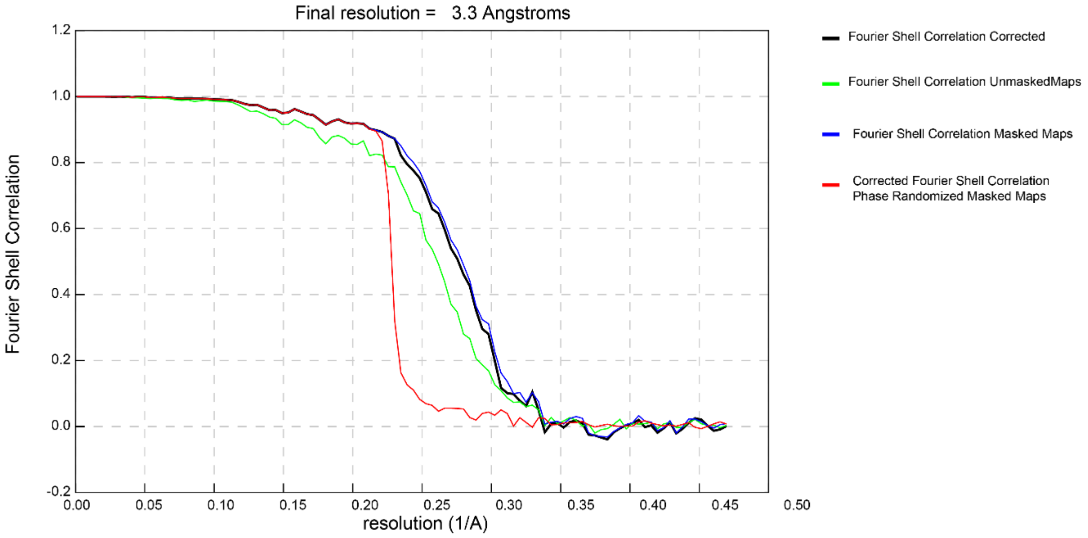

Figure S4: Fourier shell correlation plot for the cryo-EM structure of AltMV. The plot shows that the resolution that corresponds to an FSC coefficient of 0.143 is 3.3 Å.

Table S1. Primers used in cloning and RNA probe synthesis

Primer name	Sequence 5′ to 3′[1]	Description
AltMV-CP-Stop-XhoI-R	TAGCTCGAG**CTA**CTCCGGTGGTGGGAGGTATTG	Introduce XhoI site downstream of AltMV-CP
pMA-tHB5'	GTAATACGACTCACTATAGGGCGAATTGGCG	Amplify genes from GeneArt® pMA plasmid
AltMV-CP-AscI-F	TCAGGCGCGCC*ATG*TCCACTCCATTTCCTCAAGTC	Introduce *AscI* site upstream of AltMV-CP
AltMV-CP-wt-XmaI-R	ACACCCGGG**CTA**CTCCGGTGGTGGGAGGTATTG	Introduce XmaI site downstream of AltMV-CP
PapMV-CP-AscI-F	TCAGGCGCGCC*ATG*TCTAAGTCTTCAATGTCTACTCCTAATATTGC	Introduce *AscI* site upstream of PapMV-CP
PapMV-CP-wt-XmaI-R	ACACCCGGG**TTA**TTCCGGTGGAGGAAGAAATTG	Introduce XmaI site downstream of PapMV-CP
CVYV-CP-AscI-F	TCAGGCGCGCC*ATG*GCAGACGACATTGAGAAAGAAG	Introduce *AscI* site upstream of CVYV-CP
CVYV-CP-XmaI-R	ACACCCGGG**TCA**TTCAATCATCGCTCCACCATAAG	Introduce XmaI site downstream of CVYV-CP
SPFMV-CP-AscI-F	TCAGGCGCGCC*ATG*TCTAGTGAACGTACTGAATTCAAAGATG	Introduce *AscI* site upstream of SPFMV-CP
SPFMV-CP-XmaI-R	ACACCCGGG**CTA**TTGCACACCCCTCATTCCTAAGAG	Introduce XmaI site downstream of SPFMV-CP
TMV-CP-AscI-F	TCAGGCGCGCC*ATG*TCTTACAGTATCACTACTCCATCTCAGTTCG	Introduce *AscI* site upstream of TMV-CP/OAS
TMV-OAS-XmaI-R	ACACCCGGGCCTAGGCCGGTTCGAGATCG	Introduce XmaI site downstream of TMV-CP/OAS
AltMV-probeF1	CAGTTGACCACCATTGCTAGTC	Synthesis of RNA probe for anti-AltMV-CP northern blot
AltMV-probeR1	taatacgactcactatagggGGCAAAGAATCTGCAGAATTTG	Synthesis of RNA probe for anti-AltMV-CP northern blot
PVX-probeF1	CTCACAGAGATCACAACGGAAAC	Synthesis of RNA probe for anti-PVX Rep northern blot
PVX-probeR1	taatacgactcactatagggATGGTTGCCTGGTATATACTGGAAAC	Synthesis of RNA probe for anti-PVX Rep northern blot

[1] Restriction sites are underlined. *Start codons* are in italics. **Stop codons** are in bold. T7 promoter sequence is lower case.

Table S2. Cryo-EM data collection, refinement and validation statistics

	AltMV
**Data collection and processing**	
Sample applications to grid	1
Magnification	75, 000 x
Voltage (kV)	300
Electron exposure (e–/Å^2)	74.26
Defocus range of micrographs (μm)	-0.5 to -2.0
Pixel size (Å)	1.065
Symmetry imposed	Helical
Initial particle images (no.)	388, 226
Final particle images (no.)	32, 018
Map resolution (Å)	3.3
FSC threshold	0.143
Number of frames	59
**Refinement**	
Map sharpening *B* factor (Å^2)	-170.4
Model composition	
Protein residues	199
Nucleic acids	5
R.m.s. deviations	
Bond lengths (Å)	0.007
Bond angles (°)	0.655
**Validation**	
Clashscore	7.29
Poor rotamers (%)	0.00
Ramachandran plot	
Favored (%)	90.36
Allowed (%)	9.64
Disallowed (%)	0.00
